# The Development of Spinal Deformity in Patients with Duchenne Muscular Dystrophy: Clinical Assessment, Surgical Considerations and Recommendations for Treatment

**DOI:** 10.3390/jcm15062116

**Published:** 2026-03-10

**Authors:** Athanasios I. Tsirikos, Simon B. Roberts

**Affiliations:** Scottish National Spine Deformity Centre, Royal Hospital for Children and Young People, 50 Little France Crescent, Edinburgh EH16 4TJ, UK; simon.roberts@nhs.scot

**Keywords:** Duchenne muscular dystrophy, scoliosis, surgical, functional, outcomes

## Abstract

Duchenne muscular dystrophy (DMD) causes progressive skeletal, respiratory and cardiac muscle weakness in affected males. Most DMD patients develop scoliosis following loss of ambulation. This narrative review describes recommendations for the management of scoliosis in DMD patients using a review of the current literature evidence and a consensus review by the DMD Care UK Spinal Surgery Working Group. Advances in medical treatments have improved life expectancy for DMD patients. Spinal bracing is not effective in preventing the deterioration of scoliosis. Seating and wheelchair adaptations can provide postural support. The multidisciplinary assessment of patients with DMD requiring treatment for scoliosis is reviewed, with particular focus on bone, cardiac and respiratory health. The indications, surgical techniques, and type of spinal instrumentation for surgical management for progressively severe scoliosis with or without pelvic obliquity are discussed. Anaesthetic techniques, intraoperative neuromonitoring, perioperative care, and postoperative management in the ICU are discussed for the optimal management of DMD patients undergoing surgery to correct spinal deformity. Finally, regional and holistic functional assessments, patient satisfaction and long-term health, quality of life, and life expectancy for DMD patients undergoing treatment for spinal deformity are reviewed.

## 1. Introduction

Duchenne muscular dystrophy (DMD; Online Mendelian Inheritance in Man [OMIM] reference 310200) is an X-linked recessive disease with an incidence of 1 in 5000 live male births and may result in death from respiratory failure in late adolescence or early adulthood in the absence of modern medical treatment [1]. Mutations in the *DMD* gene located at Xp21.2-21.1 lead to an absence or defect in the protein dystrophin resulting in skeletal, respiratory, and cardiac muscle weakness [2,3,4]. The dystrophin protein provides a mechanical link between cytoskeletal actin and the extracellular matrix, and protects the sarcolemma during stretch and contraction; in muscle cells dystrophin thereby functions to maintain membrane integrity and prevent membrane rupture [5]. The natural history of untreated DMD leads to progressive muscle weakness with a loss of ambulatory ability and wheelchair dependence occurring most commonly at around 10–12 years of age [6]. Scoliosis develops in DMD patients following the loss of ambulatory function [3,7,8,9,10,11,12,13,14,15,16,17,18]. This is associated with a worsening vital capacity due to progressive muscle weakness [19]. Variable phenotypic expression of the disease is due to the type of mutation and related dystrophin expression. Current evidence has not consistently demonstrated that specific genetic variants causing DMD lead to a more severe progression of spinal deformity [20,21,22].

Improvements in medical treatments have prolonged the life expectancy for DMD patients [23,24]. Use of corticosteroids, as well as respiratory, cardiac, orthopaedic, and rehabilitative treatments have improved the function, quality of life, health, and life expectancy for patients newly diagnosed with DMD. DMD patients who have not received steroid therapy have a high risk of developing significant progressive scoliosis, with rates approaching 90% by skeletal maturity, most of whom will require scoliosis surgery [25]. In contrast, long-term steroid therapy delays the loss of ambulation by several years, reduces the prevalence (31% compared to 91% prevalence) and severity of scoliosis, and decreases the need for spinal surgery (8–22% compared to 78–92%) [26]. The onset of scoliosis is delayed, and the progression is slower in steroid-treated DMD patients, with many not developing significant spinal deformity even after 10–15 years of steroid therapy [26,27,28].

Several new therapies are being investigated to modify the disease course for DMD patients, though drug approval and access varies across countries [29]. For patients with specific genetic mutations, exon skipping therapies have been developed that may restore partial dystrophin function. Stop codon read-through therapy is conditionally available in the UK for patients with nonsense mutations. Gene therapy with microdystrophin also remains under investigation. Givinostat, a histone deacetylase inhibitor approved for treatment of DMD patients who are over 6 years of age, has shown efficacy in delaying disease progression regardless of genotype. Other disease-modifying therapies, such as anti-inflammatory agents and NF-κB inhibitors are a major focus of research for treating DMD.

Spinal bracing is not effective in preventing the deterioration of scoliosis in DMD patients [30]. Seating adaptations, such as anterior tilt, may improve spinal stability by promoting lumbar lordosis, and may delay the onset of spinal deformity [31]. Seating adaptations also provide postural support and improved sitting balance. Custom-moulded seating may reduce the rate of progression of scoliosis, compared to modular seating [25]; however, scoliosis continues to deteriorate and custom-moulded seating may be more amenable once the patient has completed their spinal growth. Seating adaptations fitted in the wheelchair can provide postural support and improve sitting balance at the early stages of curve development but do not prevent the deterioration of scoliosis with continued spinal growth. Surgical intervention to correct spinal deformity is indicated when progressive scoliosis develops. The aim of surgical intervention in these patients is to achieve and maintain good sitting balance by correcting the spinal deformity and associated pelvic obliquity so that wheelchair sitting and posture are comfortable for the patient [32]. Several studies have reported high complication rates associated with surgery to correct spinal deformity in DMD patients. Patients with DMD are at risk of adverse reactions to anaesthetic medications, high-volume blood loss, prolonged intensive care unit (ICU) admission, tracheostomy, respiratory infection, ventilator dependency, wound and soft tissue compromise, and instrumentation failure [19]. The progression of the underlying disease results in the loss of independent ambulation, and subsequently the development of scoliosis, respiratory failure, and cardiomyopathy, with a median life expectancy of 28.1 years for patients born after 1990 (95% CI 25.1, 30.3) [23,33].

The aim of this review is to present recommendations for the management of scoliosis in patients with DMD based on the consensus review by meetings of the DMD Care UK Spinal Surgery Working Group between 1 April 2024 and 22 May 2025; the group comprised consultant orthopaedic spinal surgeons, the DMD Care UK manager and coordinator, parents and care givers, paediatric and paediatric neuromuscular physiotherapists, and consultants in neurology, neuromuscular diseases, respiratory medicine, and paediatric medicine. The literature search was completed in March 2024 and updated December 2025, comprising searching PubMed/MEDLINE for publications with terms “Duchenne muscular dystrophy” OR “DMD” AND “scoliosis” OR “spinal deformity” OR “kyphosis” OR “Cobb angle” OR “pulmonary function” OR “functional outcomes” AND “surgery” OR “scoliosis surgery” OR “spinal surgery” OR “spinal fusion” OR “conservative” OR “nonoperative” OR “bracing” OR “treatment outcomes”. The recommendations are intended for healthcare professionals involved in the multidisciplinary care of DMD patients, with a focus on the treatment of spinal deformity.

## 2. Development of Scoliosis

All DMD patients should be monitored clinically for the development of scoliosis as part of every clinical examination. This may be by visual inspection by experienced clinicians. Spinal radiographs may be helpful when an inspection is not informative, such as in children and adolescents who are very overweight. Spinal radiographs are indicated to assess spinal deformity if there is clinical suspicion of spinal deformity, or as a baseline assessment around the time of a patient becoming wheelchair-dependent. Whole spine radiographs in the coronal and sagittal planes are required. Skeletally immature DMD patients with spinal deformity should undergo spinal radiographs every 6 months, and skeletally mature DMD patients should undergo spinal radiographs at least once each year. The referral to an orthopaedic spinal surgeon for clinical review should be performed when a DMD patient develops a scoliosis greater than 20° Cobb angle [34].

Patients with DMD typically develop a long C-shaped collapsing scoliosis of the thoracic and lumbar spine with the apex in the thoracolumbar junction [35]. Alternatively, they may develop a primary lumbar and secondary thoracic scoliosis. The scoliosis develops due to inherent progressive trunk muscle weakness and the inability of the upper body to withstand the gravity effect during phases of rapid skeletal growth. The pelvis is often tilted on the concave side of the scoliosis and becomes part of the deformity further affecting the ability of the patient to achieve comfortable seating balance. Costo-pelvic impingement with the lower concave ribs resting against the elevated iliac crest can result in localised pain which is aggravated in the sitting position. Thoracolumbar kyphosis can also develop alongside scoliosis, and this can cause back pain while further compromising the patient’s posture and trunk alignment [35].

Older studies reported at least 85% of DMD patients to exhibit a mean rate of scoliosis progression of 2.1° per month [16,36]. Forced vital capacity (FVC) declines at a rate of 4% each year after the loss of independent ambulation, and a further 4% decline is associated with each 10° of scoliosis deterioration [37]. Maintaining ambulatory function for as long as possible with the use of corticosteroids can reduce the risk of the development of scoliosis and can decrease the severity of scoliosis in the long term. The rate of scoliosis deterioration and pulmonary compromise has decreased in recent years in the advent of corticosteroid treatment using daily regimens. Alternate steroid regimens may be considered and require discussion with the patient and their families regarding their preferences, and the regimen efficacy and side-effects profile [38]. The consequent pelvic obliquity together with weakness of the spinal musculature impairs patients’ sitting posture in a wheelchair [30]. Spinal stabilisation is recommended to correct the spinal deformity and pelvic obliquity, and to also prevent a further progression of scoliosis. It has been demonstrated that surgical correction of scoliosis improves the sitting ability and quality of life of DMD patients [19,39,40].

## 3. Indications for Scoliosis Correction

Severe spinal deformity in patients with DMD can lead to poor sitting balance, back pain, difficulty in providing personal care, and poor body image. Patients become wheelchair-dependent at a mean age of 10–12 years, and develop significant scoliosis at 13–15 years of age. The rapid progression of scoliosis with associated pelvic obliquity and poor sitting posture when using a wheelchair develops from the age of 15 years onwards. Surgical correction of spinal deformity can be considered in non-ambulatory DMD patients with a progressive scoliosis greater than 20–30° in the sitting position, though the risks and benefits of surgery and the functional impact of surgery for each patient must be carefully discussed [34]. Scoliosis surgery can achieve a significant correction of the curve with a limited loss of correction over time [41]. Early surgery may cause a height restriction from premature growth arrest. This can be of concern as trunk height is also caused by pubertal delay that often occurs in patients DMD; this is where testosterone treatment may have a role to stimulate skeletal growth and improve bone health. 

Previous reports suggested that DMD patients undergoing correction of scoliosis for a spinal curvature < 40° obtained better immediate correction, which remained stable over time [41]. Prophylactic or early surgery has been previously recommended, when the scoliosis reaches 10–40°, and vital capacity is >35–40% predicted [41]. Some surgeons have recommended timing scoliosis surgery on the basis of each patient’s cardiac condition rather than their respiratory health [41]. Lumbar lordosis is important for a comfortable sitting balance in DMD patients, especially as flexion contractures of the lower limbs are usually present [30]. Superior correction of spinal deformity has been reported with the use of screw-based sacropelvic fixation compared to the traditional Galveston technique of intra-iliac rod stabilisation that has been largely abandoned (60% versus 33% correction rates) [42]. Satisfactory pelvic balance may be restored without any disadvantages or problems arising from instrumentation to the pelvis [30].

Posterior instrumented spinal fusion is the optimal surgical intervention for progressive scoliosis in DMD patients [3,40,41,43]. Spinal stabilisation is recommended before the deterioration of cardiac and respiratory function precludes safe general anaesthesia, and while the scoliosis remains flexible [11,16,44,45,46]. Cardiac MRI has added value over echocardiography in the identification of DMD-related cardiomyopathy [47,48]. This provides a detailed structural and functional assessment of the heart. It is particularly effective in clarifying equivocal findings and for those patients in whom reliable cardiac measures cannot be obtained by ultrasound. However, cardiac MRI can be challenging in patients with DMD who have behavioural issues (and may not tolerate it without the need for general anaesthetic), are significantly obese or are claustrophobic. Modern spinal instrumentation to correct scoliosis is titanium-made which is compatible with MRI; however, the images can be partly distorted by artefacts.

## 4. Preoperative Assessment Before Scoliosis Surgery

Surgical treatment of scoliosis in DMD patients can only be performed in tertiary centres where extensive medical support, anaesthetic expertise and an ICU are available. In addition, spinal surgeons involved in the care of this group of patients should have an established regular practice that treats neuromuscular scoliosis so they develop competence in assessing patients with DMD, deciding on the indications for surgery and being familiar with the surgical techniques that are required in the treatment of this type of complex spinal deformity. Close collaboration with other specialties providing care for these children is essential to define their management priorities and coordinate multi-faceted treatments in order to effectively address their needs at different stages of their life.

A multidisciplinary team (MDT) preoperative evaluation is required to assess patient co-morbidities before a decision is made to proceed with scoliosis correction. This can be planned in a dedicated multi-speciality pre-assessment anaesthetic-led clinic that involves overnight sleep studies followed by anaesthetic, respiratory, cardiology, dietician/gastroenterology, neurology, physiotherapy and occupational therapy reviews, as well as baseline blood tests. An MDT meeting between the surgical team and the medical/allied healthcare professional (AHP) colleagues is scheduled once the assessment is complete; this focuses on a discussion around the particular needs of the individual patient to determine the anticipated benefits of surgery, and the risks involved before a final recommendation is made to the family regarding treatment. This holistic approach also gives the opportunity to optimise the patient’s co-morbidities and provides adequate preparation for spinal surgery. This includes establishing non-invasive ventilation if this is required, treating cardiac dysfunction, providing nutritional support, and making provision for equipment at home (i.e., a hoist) or housing adaptations needed when the patient is discharged after surgery.

When the risks of complications associated with scoliosis surgery are discussed with the patients and their families these should include the need for prolonged postoperative invasive and non-invasive ventilation, the possibility of requiring a tracheostomy, the risk of major cardiac complications related to pre-existing reduced cardiac contractility and arrhythmias that can cause rapid patient decompensation during induction of anaesthesia or as a result of major blood loss and hypovolemia, and the risk of death.

## 5. Posterior Instrumented Fusion for Scoliosis Correction

Scoliosis surgery in the context of DMD patients has considerably improved in the last 40 years. This change can be attributed to the development of advanced surgical techniques, the advent of modern spinal instrumentation, and the significant improvements in preoperative assessment and perioperative MDT management of this group of patients.

### 5.1. Type of Approach for Scoliosis Correction

Patients with DMD and severe spinal deformity are treated by a posterior spinal correction and fusion. The aim of the surgery is to produce a balanced spine in the frontal and the lateral planes with the head centred above the pelvis while achieving a solid fusion that can avoid implant failure resulting in a recurrence of the deformity and necessitating revision surgery. The addition of an anterior spinal release is not recommended in the presence of an underlying respiratory compromise that can significantly increase the risk of major complications, and also lead to prolonged surgical time and intraoperative blood loss.

### 5.2. General Considerations

Patient positioning in the surgical bed can be challenging due to upper/lower limb contractures and pelvic deformity. Any bony prominences need to be well-padded to prevent the development of pressure sores. Avoiding pressure to the abdomen is essential to reduce intraoperative blood loss with consequent hypovolemia and the development of coagulopathy. The use of tranexamic acid and cell-salvage along with hypotensive anaesthesia and prompt correction of coagulopathy using fresh frozen plasma (FFP) and platelets can decrease blood loss during surgery. Despite all these efforts blood transfusion is often required intraoperatively and/or postoperatively [49].

The anaesthetist involved in the care of DMD patients undergoing scoliosis correction should have a thorough understanding of the associated medical problems related to this condition and their management in the context of major surgery. Most DMD patients are prescribed corticosteroids and are therefore at increased risk of adrenal insufficiency. In these patients, corticosteroid replacement is a critical consideration in their perioperative management. Total Intra-Venous Anaesthesia (TIVA) is the recommended anaesthetic approach as the use of succinylcholine and volatile anaesthetics (such as isoflurane) increases the risk of developing severe hyperkalaemia and anaesthesia-induced rhabdomyolysis (AIR). The depolarizing muscle relaxant suxamethonium should not be used in DMD patients as it is associated with AIR and the risk of hyperkalaemic cardiac arrest [34].

The use of neuromonitoring is essential during surgical intervention to correct scoliosis in order to reduce the risk of neural damage during instrumentation placement or while deformity correction manoeuvres are applied, as patients with DMD retain their bladder/bowel control and sensory function. This focuses on recording cortical and cervical somatosensory evoked potentials.

### 5.3. Type of Spinal Instrumentation—Evolution of Implants

Sublaminar wire instrumentation was popularised in the 1980s for the treatment of neuromuscular scoliosis and this was applied in the correction of scoliosis for DMD patients. Currently, sublaminar wire instrumentation is not commonly used to treat scoliosis in the advent of 3rd generation segmental pedicle screw implants. When used in the lumbar spine, sublaminar wires result in higher rates of implant failure [7]. In contrast, lumbar fixation using pedicle screws can produce improved spinal stability and avoid the need for instrumentation to the pelvis in the presence of mild pelvic obliquity (<15°). Pedicle screw fixation over several levels of the thoracic and lumbar spine provides sufficient mechanical stability to correct the spine in a balanced upright position, and indirectly allows for the partial correction of pelvic obliquity by distracting the concavity of the scoliosis across the lumbar segments [35].

In previous reports, spinal instrumentation with pedicle screws for scoliosis correction in DMD patients has resulted in the effective correction of both spinal and pelvic deformity [30]. A pelvic obliquity correction of 41.5% has been achieved with the use of pedicle screw constructs without fixing the rods distally to the pelvis. However, it should be noted that if the spinal fusion has spared the lumbosacral junction, pelvic obliquity may increase postoperatively (with an incidence of up to 84%) in skeletally immature patients due to remaining growth. Despite this, patients do maintain their sitting balance and usually do not require revision surgery.

In addition, rigid pedicle screw instrumentation provides sufficient mechanical stability to allow DMD patients to mobilise immediately postoperatively without the need for external trunk support, which is very important for DMD patients to avoid respiratory complications in the immediate postoperative period [30]. The correction of lumbar sagittal balance from 15.6° of kyphosis to 22.4° of lordosis has been reported with the use of pedicle screw instrumentation [50]. Flexion contractures of the hips and knees, which are often present in DMD patients, make restoration of lumbar lordosis essential for these patients in order to achieve comfortable and balanced sitting control [30]. Pedicle screw constructs permit a good reconstruction of lumbar lordosis [51]. Spinal instrumentation of DMD patients to L5 has been reported to enable restoration of lumbar lordosis from 20° preoperatively to 42° postoperatively [52]. This has the advantage of preserving mobility at the L5-S1 level that may be beneficial for sitting and transfer activities; a distal fusion level of L5 may only be valid if the curve apex is at L2 or higher and if the L5 tilt is <15°. Segmental pedicle screw instrumentation and fusion to L5 permit a stable long-term correction of scoliosis and pelvic obliquity.

Another advantage of the surgical correction of scoliosis with instrumentation involving only pedicle screws is that these are made of titanium, which reduces the risk of infection compared to stainless steel implants such as sublaminar wires; titanium has self-oxidising properties that provide resistance to bacterial colonisation. The majority of spinal implants for scoliosis correction, particularly titanium and titanium alloy implants, are regarded as MR-conditional and safe for postoperative MR imaging. Cardiac MRI following posterior instrumented spinal fusion may be impaired if the spinal instrumentation is within or adjacent to the cardiac imaging field, especially in the thoracic spine; the diagnostic quality for cardiac MR may be compromised [53,54,55]. Carbon fibre spinal instrumentation that offers minimal MR imaging artefacts are not accepted as standard of care for scoliosis surgery [56]. However, advanced metal artefacts reduction MR imaging techniques and lower field strengths can mitigate against spinal instrumentation artefact severity and may offer a more amenable strategy for cardiac imaging following posterior instrumented spinal fusion for spinal deformity in DMD patients [54,55,57].

### 5.4. Comparison of Types of Instrumentation Techniques

Different types of instrumentation constructs for the correction of scoliosis (such as sublaminar instrumentation, hybrid instrumentation with sublaminar wires and pedicle screws, and all-pedicle screw constructs) in DMD patients have been compared [58]. Each instrumentation construct provided and maintained the correction of scoliosis during long-term follow-up. However, the correction of scoliosis with pedicle screws, and without extending instrumentation to the pelvis, is the preferred technique as it is associated with less blood loss and shorter surgical time. The correction achieved by hybrid instrumentation and all-pedicle screw constructs is greater than that when sublaminar wire instrumentation is used. When accounting for preoperative curve flexibility, pedicle screw constructs provide better correction and maintain correction more effectively at final follow-up.

Hybrid constructs using thoracic sublaminar wires and lumbar pedicle screws have previously been utilised in DMD patients. Although a satisfactory coronal deformity correction of 41–66% has been achieved using hybrid instrumentation techniques, the rate of complications is high including a 21% incidence of implant-related problems and 10% incidence of pseudarthrosis [59]. Junctional kyphosis and progressive thoracic kyphosis have also been described in up to 22% of patients treated with hybrid instrumentation due to the reduced stiffness of sublaminar wires used for instrumentation of the thoracic spine [60,61,62]. Pedicle screws provide three-column control and rigid constructs to achieve and maintain adequate deformity correction compared to sublaminar wires [63].

Pedicle screw instrumentation is better than sublaminar wire instrumentation for the correction of coronal curvature (83% versus 62% correction rates) and pelvic obliquity in DMD patients with scoliosis; satisfactory sitting balance is restored without the need for pelvic or sacral fixation in patients with pelvic obliquity < 15° [64]. However, in the presence of more severe pelvic tilt or in skeletally immature patients the extension of instrumentation to the pelvis maintains better correction of pelvic obliquity in comparison to instrumented fusions terminating more proximally. In addition, pelvic fixation may avoid, in young patients with remaining growth progression and progressive pelvic obliquity, the risk of needing further revision surgery to extend the fusion at a time when the health of these patients may be deteriorating [65].

### 5.5. Levels of Fusion

**Figure 1:**
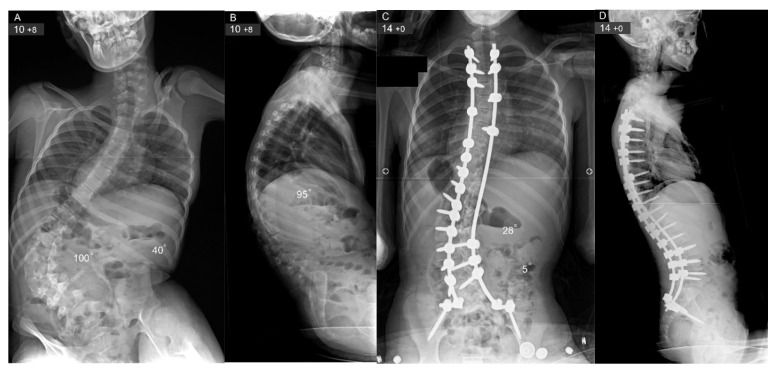
Patient with DMD who lost his ambulatory function at a young age and developed a severe collapsing kyphoscoliosis with marked pelvic obliquity (**A**,**B**). The patient underwent a posterior spinal fusion extending from T2 to the sacrum with pelvic fixation of the rods at age 10 years and 8 months. This resulted in an excellent correction of the kyphoscoliosis and a balanced spine in the coronal and sagittal planes with a level pelvis (**C**,**D**).

There is a limited consensus in regard to the optimal caudal extent of spinal instrumentation in the surgical management of scoliosis in DMD patients [66]. Traditional treatment recommendations suggested extending the instrumentation distally to the sacrum or pelvis (Figure 1) with the aim of correcting the associated pelvic obliquity and restoring sitting balance of the trunk [66]. However, extending instrumentation to the sacropelvis is associated with a higher rate of complications [66]. Pelvic fixation has a higher complication rate as it prolongs surgery leading to increased blood loss, and it is also associated with a greater risk of wound infection as the skin incision is closer to the anus and can be contaminated [66]. Posterior instrumented spinal fusion with distal fixation to L4 or L5 has been recommended in patients with milder scoliosis without significant pelvic obliquity, whereas fixation to the sacropelvis may be reserved for larger curves with more severe pelvic obliquity (>15°) [40,67]. The decision whether or not to extend the instrumented spinal fusion to the distal lumbar spine (L4/5) or sacropelvis must take into consideration each patient’s medical comorbidities and ability to tolerate extensive surgery, lower trunk soft tissue health, and the patient’s preoperative satisfaction with their sitting balance. Previous comparisons between terminating instrumented fusions at the lower lumbar spine or pelvis have led to recommendations for the use of instrumentation and fusion to L5 when the Cobb angle was <40° and pelvic obliquity < 15° [12]. Sacropelvic fixation may be considered when the apex of the spinal curvature is below L2.

## 6. Postoperative Management After Scoliosis Surgery

Immediately following the completion of corrective scoliosis surgery, DMD patients are transferred to the ICU for postoperative care that includes haemodynamic support, non-invasive ventilation, intense respiratory physiotherapy, early mobilisation using their wheelchair and nutritional supplementation through nasogastric feeding. These patients are discharged from the ICU to the surgical ward when they are medically stable. These patients are then discharged from the surgical ward to return home when oral nutrition, pain control, respiratory function and wound healing are well-established.

## 7. Complications Associated with Scoliosis Correction

Complications of scoliosis surgery in DMD patients include large-volume intraoperative blood loss, postoperative infection, and respiratory or feeding problems [11,13,19,39,68]. Patients with DMD have been reported to have higher blood loss at surgery to correct spinal deformity, compared to other cohorts of patients undergoing corrective scoliosis surgery [69]. There is no evidence that disease progression, as measured by respiratory function, leads to greater blood loss. A higher incidence of deep surgical site wound infections postoperatively has been described in DMD patients than in patients with other neuromuscular conditions [70]. An increased incidence of complications has been reported for DMD patients with FVC less than 30% compared to those with FVC greater than 30% [19,70]. Hepatotoxicity has been described in patients with DMD after scoliosis surgery compared to other neuromuscular conditions [70]. Risk factors for an extended patient stay in the ICU postoperatively include a larger preoperative scoliosis angle and greater intraoperative estimated blood loss during surgery, as well as the development of cardiopulmonary complications [71].

## 8. Current Surgical Considerations Around Scoliosis Correction

Historically, children with DMD would be offered surgery once they lost their walking ability and as soon as they developed a mild scoliosis of even 10–20°. This notion was based on the anticipated deterioration in the degree of the curve that was expected to occur with further spinal growth and the impact that the scoliosis could have on the patient’s respiratory function. The surgery traditionally involved an extensive fusion from the upper thoracic to the low lumbar spine or the sacrum/pelvis. This early indication for treatment resulted in the majority of patients with DMD undergoing surgery in their late child life or early teenage years. As a consequence, many patients complained of losing function after surgery despite having a stable and balanced spine, as they could not bend over and use their hands close to their face for the activities of daily life, such as to feed themselves or use portable digital devices. On occasions this restriction in trunk movement was associated with patients’ dissatisfaction regarding the outcomes of surgery. Early surgical correction of scoliosis is essential in DMD patients who either have a rapid progression of their scoliosis, or whose respiratory and cardiac function are deteriorating to the extent that major surgery to correct their scoliosis cannot be performed safely at a later time.

**Figure 2:**
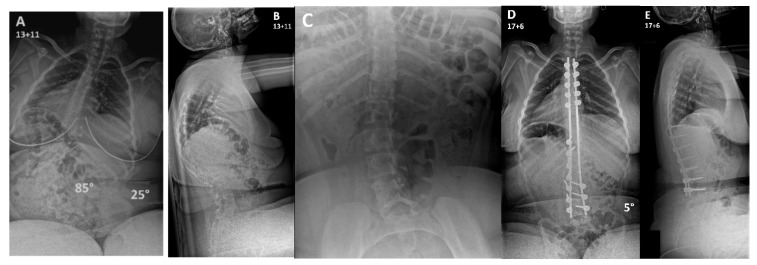
Patient with DMD who developed a severe C-shaped kyphoscoliosis (**A**,**B**). On a preoperative supine traction view the scoliosis and associated pelvic obliquity retained flexibility (**C**). This allowed the patient to undergo a posterior spinal fusion extending from T2-L4, which preserved distal spinal mobility while achieving excellent deformity correction in both planes with a level pelvis (**D**,**E**).

In the current era of modern management of DMD, early treatment with corticosteroids has reduced the development of severe scoliosis in these patients to the extent that the number of patients needing surgical correction has significantly decreased. It is, therefore, imperative that any decision regarding scoliosis surgery takes into consideration the individual patient’s needs and their symptoms due to scoliosis, and not focus primarily on the radiographic severity of scoliosis. As an example, patients who develop moderate lumbar curves that do not cause back pain and do not impact on their sitting balance at the end of spinal growth may be left untreated to preserve their functional skills as this type of scoliosis does not impact on pulmonary function. In contrast, rapidly progressive scoliosis that leads to deterioration in pulmonary function requires consideration of early surgical treatment in order to prevent further respiratory decline. In addition, when scoliosis surgery is required it is recognised that it is advantageous to preserve distal spinal flexibility by stopping the fusion ideally at L4 or at least at L5 (Figure 2).

Another important consideration that needs to be brought to the patients’ and their families’ attention is the fact that major spinal surgery will require the instigation of non-invasive ventilation (NIV) during the postoperative period for patients who may have not previously needed it. After scoliosis correction these patients often stay on overnight ventilatory support including NIV for the rest of their life.

## 9. Respiratory Function After Surgical Correction of Scoliosis

The overall evolution of the restrictive respiratory disease in patients with DMD is not modified by the surgical correction of scoliosis [41]. Respiratory surveillance may consist of the measurement of FVC at 6-monthly intervals, supplemented by symptom assessment and home overnight pulse oximetry once FVC falls to <1.25 L [23,72]. Indications for ventilation include development of symptomatic nocturnal hypoventilation or a FVC < 0.6 L [23]. The loss of vital capacity after the operation is mild. The rate of the loss of vital capacity during postoperative follow-up has been reported to be comparable to that of the natural history of the condition [41]. Patients’ vital capacity following scoliosis surgery was not significantly different in those patients undergoing surgery for a scoliosis angle <40° compared to patients undergoing surgery for a scoliosis angle > 40° [41]. Respiratory function may stabilise for up to 3 years after scoliosis correction [9,44]. A preoperative forced expiratory volume in one second (FEV1) < 40% (of predicted value) has been associated with the development of postoperative respiratory complications [73]. FVC of <35% is associated with a greater incidence of pulmonary complications postoperatively [11].

Respiratory function in DMD patients declines most rapidly during the adolescent growth spurt; FVC declines to 35% at a mean age of 15 years [74]. FVC decreases by 4% each year, and/or each 10° of scoliosis progression [37]. FVC < 35% in DMD patients has been previously reported to be a relative contra-indication for scoliosis surgery [18,37]. Previous studies have not shown a significant effect of scoliosis surgery on subsequent mortality in DMD patients [3,41,74]. This is not surprising as most studies have failed to show a protective role of spinal surgery on FVC in DMD patients [3,74,75]. Following scoliosis surgery, there is a reported 7% fall in FVC [76].

Despite a lack of consensus on the effect of posterior instrumented spinal fusion to correct scoliosis on the decline of respiratory function in DMD patients, spinal fusion does still provide improved sitting balance, improved pain and discomfort, easier nursing care, and improved quality of life [3,40,41,43]. There is no significant correlation between the severity of scoliosis and respiratory dysfunction; this may be because scoliosis only negatively impacts respiratory function when the curve angle exceeds 50–60° [77,78].

Scoliosis surgery improves the rate of respiratory function deterioration in DMD patients from an FVC decline of 8% per year preoperatively, to 3.9% per year postoperatively [44]. Similar trends have been reported in other studies with the rate of decline in FVC reported as 4% per year preoperatively, which was reduced to 1.75% per year after surgery [44]. FVC values decreased in DMD patients regardless of whether or not they underwent surgical treatment for scoliosis, but the mean rate of deterioration was significantly less in the surgical group [79]. A reduced rate of decrease in FVC, reduced dependence on non-invasive ventilation, and higher reported scores on the Muscular Dystrophy Spine Questionnaire (MDSQ), together with patients’ subjective reports that breathing is easier, indicate that spinal surgery might decrease the rate of deterioration of respiratory function [79]. Some studies have reported that surgical treatment for DMD scoliosis increases FVC for several years, delays the decline of respiratory function, and improves the subsequent survival rate [80].

These outcomes have been reported in DMD patients and FVC < 30% undergoing surgery: patients with high-risk respiratory dysfunction and severe scoliosis could undergo scoliosis correction with pedicle screw instrumentation after respiratory muscle training. All-pedicle screw instrumented spinal fusion achieved the correction of spinal deformity in the coronal and sagittal planes and correction of pelvic obliquity, which were maintained at long-term follow-up. The mean rate of FVC decline after surgery was 3.6% per year with most patients and parents being highly satisfied as they considered scoliosis surgery improved their function, sitting balance and quality of life despite the high risk of severe complications [81].

Respiratory muscle strength in DMD patients has been assessed following scoliosis surgery by posterior instrumented spinal fusion. Small improvements in maximum inspiratory pressure (MIP) at 1 and 6 months postoperatively, and a significant improvement in maximum expiratory pressure (MEP) at 6 months postoperatively, compared to preoperative values, were reported. Surgical correction of scoliosis may, therefore, improve postoperative respiratory muscle strength in DMD patients [82]. It also improves sitting balance and thoracic deformity, thereby positively influencing respiratory muscle movement. These results also suggest that respiratory muscle training could reduce perioperative complications and effectively maintain pulmonary function [82]. However, there was no significant improvement in either %VC (vital capacity) or %FVC, which supports the view that scoliosis correction does not play a role in the improvement of respiratory function.

## 10. Role of Steroids and Bone Mineral Density (BMD) Assessment

Children and adolescent DMD patients treated with steroids maintain their ambulation for longer, have a delayed onset of scoliosis, have a lower rate of progression of scoliosis, require surgical correction of scoliosis less often, and have scoliosis surgery at a later age, compared to those without steroid treatment [83]. Steroid therapy is associated with the reduced incidence of development or progression of scoliosis in DMD patients as it prolongs ambulatory ability while allowing some patients to undergo their pubertal growth spurt, which is the most risky period for curve deterioration, while being mobile. Treatment with steroids may be associated with an increased risk of vertebral and lower limb fractures, and diminished growth [27,84]. However, the benefits of steroid therapy are widely accepted to outweigh these risks in the vast majority of DMD patients who are still ambulant. After loss of ambulation, there is less evidence of ongoing benefit but the consensus is that continued corticosteroid use has a significant benefit on respiratory and cardiac function [48,72].

Children with DMD should be regularly assessed for the presence of back pain or fractures, and spinal imaging should be performed to identify vertebral fractures early [85]. Baseline blood tests should include serum calcium, phosphate, magnesium, alkaline phosphatase and parathyroid hormone. Regular assessment of calcium, vitamin D intake, serum 25-hydroxyvitamin D3 and spine bone mineral density (BD) by dual energy X-ray absorptiometry (DEXA) scan should be performed. Spinal X-rays should be obtained at 2–3 year intervals for DMD patients not receiving steroid therapy and at 1–2 year intervals for patients receiving steroid therapy. Annual spinal X-rays should be performed for patients with back pain or declining BD. Patients with clinically significant bone fragility should be assessed for treatment with bisphosphonates. Bisphosphonate therapy has a protective effect on spinal BD and vertebral morphology in DMD patients [86,87]. It can also be used as an adjunct to optimise bone quality before scoliosis correction is planned. There currently exists no consensus on the exact duration of preoperative optimisation with bisphosphonate therapy prior to scoliosis surgery, but evidence extrapolated from adult spinal deformity surgery support that at least 2 months of therapy prior to scoliosis surgery, and for 8 months postoperatively, may be beneficial [88,89].

## 11. Functional Outcomes After Scoliosis Correction

Assessment of the postoperative functional ability of DMD patients undergoing surgical correction of scoliosis has demonstrated that most patients had a major to moderate improvement in sitting balance, cosmetic appearance, functional freedom of arms, nursing care by parents, overall satisfaction and respiratory function as compared to their preoperative functional status [50]. Scoliosis surgery results in DMD patients having a stable spine and trunk and improved back pain, and patients can use transport more easily [41,50]. MDSQ scores were significantly higher for DMD patients undergoing surgical correction of scoliosis compared to those who received non-surgical management [79,90]. Following surgical correction of scoliosis, DMD patients also reported that breathing felt easier (65%), digestion of food was easier (37.5%) and trunk posture improved (37.5%).

Further tests of function include manual muscle tests (MMTs), and quantitative muscle tests [79,91,92]. The results of MMTs showed no significant difference between surgical and non-surgical groups of DMD patients with scoliosis at initial diagnosis or at follow-up. Other tests of function in neuromuscular patients include the modified Rancho scale and Swinyard scale, though the MMT and modified Rancho scale may not be useful in the functional evaluation of DMD patients with advanced scoliosis [93,94]. Activities of daily living, especially sitting ability, were markedly improved after spinal surgery in patients with DMD scoliosis [79]. The mean visual analogue pain score (VAS) improved from 6.2 preoperatively to 1.6 at 2 years postoperatively [95].

Amongst DMD patients with FVC less than 30% undergoing surgical correction of scoliosis at mean follow-up of 4 years postoperatively, 29% of patients had lost their ability to feed themselves due to weaker arms; however, all patients reported improvement in quality of life. Their coronal spinal balance was reported to be the most important parameter affecting sitting ability [81].

Long-term functional outcomes for DMD patients following surgical correction of spinal deformity have been assessed using the MDSQ [90]. Patients with DMD undergoing surgical correction of scoliosis were satisfied at long-term follow-up, reporting favourable effects on their quality of life. Scoliosis surgery improved the quality of life of DMD patients with spinal deformity and this was maintained at long-term follow-up into adult life [35].

## 12. Patient Satisfaction After Scoliosis Correction

Following surgical correction of scoliosis, 90% of DMD patients reported that they would have surgical correction of scoliosis again [41]. Provision of nursing and daily care for DMD patients was easier in 66% of patients postoperatively [41]. Carrying patients became easier and the DMD patients asked to change position at night less often following surgical correction of scoliosis. Spinal surgery also provided a more comfortable and cosmetic sitting position [23].

A previous study reporting the long-term results for DMD patients after corrective scoliosis surgery analysed clinical and radiographic factors associated with patient reported outcomes using MDSQ scores [35]. Posterior instrumented spinal fusion was associated with a satisfactory correction of spinal deformity and pelvic obliquity which was maintained at the longest reported postoperative follow-up (mean: 10.9 years). Patients who completed the MDSQ at a mean age of 25.4 years were satisfied with the results of spinal surgery and would choose surgery again if offered. Most DMD patients (87.5%) reported no severe back pain at follow-up. Their long-term functional outcomes were predicted by patient age, age at loss of ambulation, duration of follow-up, severity of residual scoliosis postoperatively, degree of scoliosis correction and lumbar lordosis. These results support the current consensus that surgical correction of spinal deformity can have positive long-term effects on quality of life and high patient satisfaction in DMD patients [35].

## 13. Life Expectancy After Scoliosis Correction

The combination of surgical correction of spinal deformity and the provision of home nocturnal ventilation has a beneficial and additive effect on prolonging the survival of DMD patients with progressive scoliosis and respiratory failure, in the absence of severe progressive cardiomyopathy [23]. Cardiomyopathy is a determining factor that increases mortality rates among these patients [30]. Surgical correction of spinal deformity and home nocturnal ventilation together improve the survival of DMD patients to a greater degree than either alone [23]. Only 10% of DMD patients do not develop a progressive scoliosis. Patient age at ventilation and whether or not spinal surgery is required correlate with the age at which DMD patients lose ambulatory ability. Therefore, those DMD patients who walk for longer, and who are ventilated at a later age, are less likely to require spinal surgery [23]. The mortality rate of DMD patients with scoliosis is significantly lower following surgical correction of spinal deformity (8.1%), compared to non-surgical management (22%), over a mean follow-up of 6.4 years [80].

In a previous study investigating the survival rates after scoliosis correction among 43 DMD patients, the median survivorship was 14 years and 2 months, with the longest observed patient survival being 22 years and 6 months [96]. Survival after scoliosis correction in this study was reported to be affected by factors that related to their underlying disease, severity of spinal deformity, and surgical morbidity. The degree of preoperative coronal imbalance and pelvic obliquity, as well as intraoperative blood loss, were factors that significantly affected the subsequent survival of DMD patients. The impact of preoperative sagittal imbalance and extension of the instrumented spinal fusion to the sacrum or pelvis (which was required in patients with severe pelvic obliquity) trended towards significance. In contrast, the age of DMD patients at time of spinal surgery, preoperative/postoperative scoliosis, thoracic kyphosis, lumbar lordosis, scoliosis and pelvic obliquity flexibility or correction indices, postoperative coronal/sagittal balance, need of preoperative non-invasive ventilation, preoperative feeding disorders, development of surgical complications, and the length of hospital and ICU stay were variables that did not affect postoperative survival in DMD patients [96].

## 14. Conclusions

In conclusion, current corticosteroid treatment protocols have reduced the prevalence of scoliosis and the severity of the developing spinal curvatures in DMD patients. At an early stage when children lose their walking ability, seating adaptations in their wheelchair can improve their trunk balance. Regular physiotherapy and hydrotherapy can maintain the range of motion of joints and prevent joint contractures. In the presence of a progressive scoliosis or kyphoscoliosis that causes back pain, affects the patient’s sitting tolerance and impacts on their respiratory function, surgical treatment may be indicated. The aim of spinal deformity surgery in DMD patients is to achieve a balanced spine in the coronal and sagittal planes over a level pelvis. Ideally, the spinal fusion should avoid, if possible, extending spinal instrumentation and fusion beyond the L4 vertebra, or at least L5 vertebra, as this will preserve distal lumbar flexibility and allow the patient to maintain functionality. The decision to proceed with scoliosis surgery should be made on the basis of the individual patient’s needs, their clinical symptoms and personal complaints, as well as their overall medical condition and severity of medical co-morbidities. Surgical correction of spinal deformity can contribute to improved survival for DMD patients. A thorough preoperative MDT review will balance the risks and benefits of surgery, which will in turn allow the patient and his family to make an informed decision as to whether or not to proceed with major spinal deformity surgery. Following this meticulous approach and in the advent of modern surgical techniques and spinal instrumentation, very satisfactory correction of the spinal deformity can be achieved. This has been associated with high patient satisfaction and excellent clinical outcomes that are maintained at long-term follow-up.

## Data Availability

No new data were created or analyzed in this study. Data sharing is not applicable to this article.

## Abbreviations

The following abbreviations are used in this manuscript:

AIR	anaesthetic-induced rhabdomyolysis
AHP	allied healthcare professionals
DEXA	dual energy X-ray absorptiometry
DMD	Duchenne muscular dystrophy
FEV1	forced expiratory volume in one second
FFP	fresh frozen plasma
FVC	forced vital capacity
ITU	intensive care unit
MDSQ	muscular dystrophy spine questionnaire
MDT	multidisciplinary team
MEP	maximum expiratory pressure
MIP	maximum inspiratory pressure
MMT	manual muscle test
MRI	magnetic resonance imaging
NIV	non-invasive ventilation
TIVA	total intravenous anaesthesia
VAS	visual analogue pain score
VC	vital capacity
UK	United Kingdom
